# Exploring Simple Particle-Based Signal Amplification Strategies in a Heterogeneous Sandwich Immunoassay with Optical Detection

**DOI:** 10.1021/acs.analchem.3c03691

**Published:** 2024-03-18

**Authors:** Daniel Geißler, K. David Wegner, Christin Fischer, Ute Resch-Genger

**Affiliations:** Division Biophotonics, Federal Institute for Materials Research and Testing (BAM), Richard-Willstaetter-Str. 11, 12489 Berlin, Germany

## Abstract

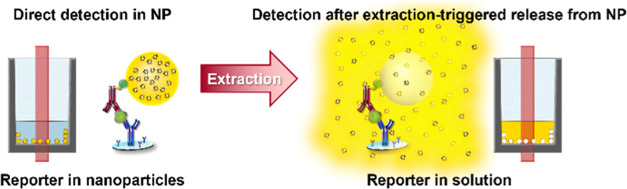

Heterogeneous sandwich immunoassays are widely used for biomarker detection in bioanalysis and medical diagnostics. The high analyte sensitivity of the current “gold standard” enzyme-linked immunosorbent assay (ELISA) originates from the signal-generating enzymatic amplification step, yielding a high number of optically detectable reporter molecules. For future point-of-care testing (POCT) and point-of-need applications, there is an increasing interest in more simple detection strategies that circumvent time-consuming and temperature-dependent enzymatic reactions. A common concept to aim for detection limits comparable to those of enzymatic amplification reactions is the usage of polymer nanoparticles (NP) stained with a large number of chromophores. We explored different simple NP-based signal amplification strategies for heterogeneous sandwich immunoassays that rely on an extraction-triggered release step of different types of optically detectable reporters. Therefore, streptavidin-functionalized polystyrene particles (PSP) are utilized as carriers for (i) the fluorescent dye coumarin 153 (C153) and (ii) hemin (hem) molecules catalyzing the luminol reaction enabling chemiluminescence (CL) detection. Additionally, (iii) NP labeling with hemin-based microperoxidase MP11 was assessed. For each amplification approach, the PSP was first systematically optimized regarding size, loading concentration, and surface chemistry. Then, for an immunoassay for the inflammation marker C-reactive protein (CRP), the analyte sensitivity achievable with optimized PSP systems was compared with the established ELISA concept for photometric and CL detection. Careful optimization led to a limit of detection (LOD) of 0.1 ng/mL for MP11-labeled PSP and CL detection, performing similarly well to a photometric ELISA (0.13 ng/mL), which demonstrates the huge potential of our novel assay concept.

Immunoassays utilizing different detection schemes are broadly applied in the life sciences as well as food and environmental analysis.^1,2^ In a conventional enzyme immunoassay (EIA) such as the current “gold standard” enzyme-linked immunosorbent assay (ELISA), the signal enhancement is achieved via the time- and temperature-dependent enzymatic generation of photometrically or fluorometrically detectable molecules.^3^ The signal size and hence the detection sensitivity can be controlled by the runtime of the signal-generating step, i.e., the enzymatic processing time of the detected substrate, rendering the influence of the spectroscopic features of the substrate almost negligible. This enzymatic signal generation concept can provide detection sensitivities in the lower μg/L range.

Although enzymatic signal generation is well established for immunoassays, many efforts have been dedicated reducing the time- and temperature-dependent signal generation steps of an EIA. This triggered the search for alternative, more simple signal generation methods that do not require an enzymatic amplification yet enable similarly good or even better detection sensitivities as an ELISA. The most popular alternative approach is fluorescence-based immunoassay (FIA) utilizing different types of luminophores for signal generation.^4−7^ For FIAs, the intensity of the measured signal depends on the brightness (*B*) of the optical reporter. *B* equals the product of the reporter’s molar absorption coefficient ε(λ_exc_) at the chosen excitation wavelength λ_exc_ and its photoluminescence (PL) quantum yield Φ_PL_.^8^ Hence, the superior brightness of many nanoparticles (NP) compared to molecular fluorophores encouraged their usage as reporters in immunoassays with optical read-out. NP explored for assay applications include nonemissive, yet strongly absorbing and/or scattering metal nanoparticles such as gold NP,^9,10^ luminescent semiconductor quantum dots, lanthanide-based upconversion nanocrystals, and fluorophore-stained silica and polymer particles, all surface functionalized with biomolecules like antibodies (AB).^11−14^ Particularly the latter two types of particle reporters, that can act as carriers for several hundreds or thousands of absorbing and/or luminescent molecules, present a simple and straightforward signal enhancement strategy^15−17^ for the subsequently detected absorption and/or PL signals.^18^ In this case, reporter brightness can be controlled by the number of absorbing and fluorescent molecules per NP and thus, also by NP size.^19,20^ For this signal enhancement strategy, *B* of the particle reporter depends on the particle size, particle matrix, and dye loading concentration, particularly for fluorophores that can interact with each other and form dye aggregates. Only for noninteracting dye molecules, as typically found for low loading concentrations, the particle’s absorption cross section σ_abs_(λ_exc_) can be directly calculated from ε(λ_exc_) of the loading fluorophore determined in a matrix modeling the polarity and refractive index of the particle matrix, and the (average) number of dye molecules per particle.^21^ These dye- and matrix-specific considerations as well as the prevention of dye-specific and concentration-dependent aggregation-induced fluorescence quenching have to be considered for the signal intensity and sensitivity optimization of FIAs using particle reporters.^6,22^

An elegant, yet rarely utilized, approach to enhance the intensity of fluorescence signals, circumventing dye aggregation-related signal losses, is the release of the signal-generating fluorescent dyes from the particles. This can be achieved by NP dissolution or by reporter extraction from the particles with a suitable solvent. After the release step, the unstained particles were removed by centrifugation, thus minimizing light scattering. Related release-based strategies have been employed for the design of fluorescent substrates for enzyme assays^23^ and so-called smart or activatable probes utilized in molecular imaging,^24,25^ particle-based FIAs with polyelectrolyte-encapsulated microcrystals of fluorescein diacetate,^18,26,27^ and sophisticated triggered release or gated systems.^28−30^ Main prerequisites to be considered for the accurate quantification with such release-based strategies are (i) the need to prevent contributions from particle size distributions on measured analyte concentration, which could otherwise introduce considerable uncertainties and blur analyte quantification, (ii) a preferably fast and quantitative release of the signal-generating species, and (iii) the minimization of unspecific interactions and uncontrolled release.

Aiming for simple enhancement strategies for FIAs, we systematically explored and compared different NP- and release-based signal amplification strategies representatively for a heterogeneous sandwich immunoassay for the detection of the inflammation marker C-reactive protein (CRP) (Scheme 1). For these proof-of-concept tests, polystyrene particles (PSP) of different size, different signal-generating cargos, and different optical detection methods were utilized. PSP were chosen as carrier beads as these polymer particles are commercially available with different surface chemistries and can be loaded with different cargos employing versatile swelling procedures.^31−33^ As signal-generating PSP cargo, (i) the fluorometrically detectable dye coumarin 153 (C153)^20^ and (ii) long-term stable hemin (hem) molecules were selected. The latter catalyze the luminol reaction, thus enabling chemiluminescence (CL) detection. Both payloads were released during the assay triggered by extraction with ethanol or by pH. In addition, (iii) the PSP carrier beads were labeled with the hemin-based catalyst microperoxidase MP11 that also catalyzes the luminol reaction. After exploring the parameters affecting signal intensities for these three amplification concepts, the performance of optimized NP reporter systems was assessed in a representatively chosen CRP assay, and the achievable analyte sensitivities and detection limits were compared with the established ELISA concept for photometric and CL detection.

**Scheme 1:**
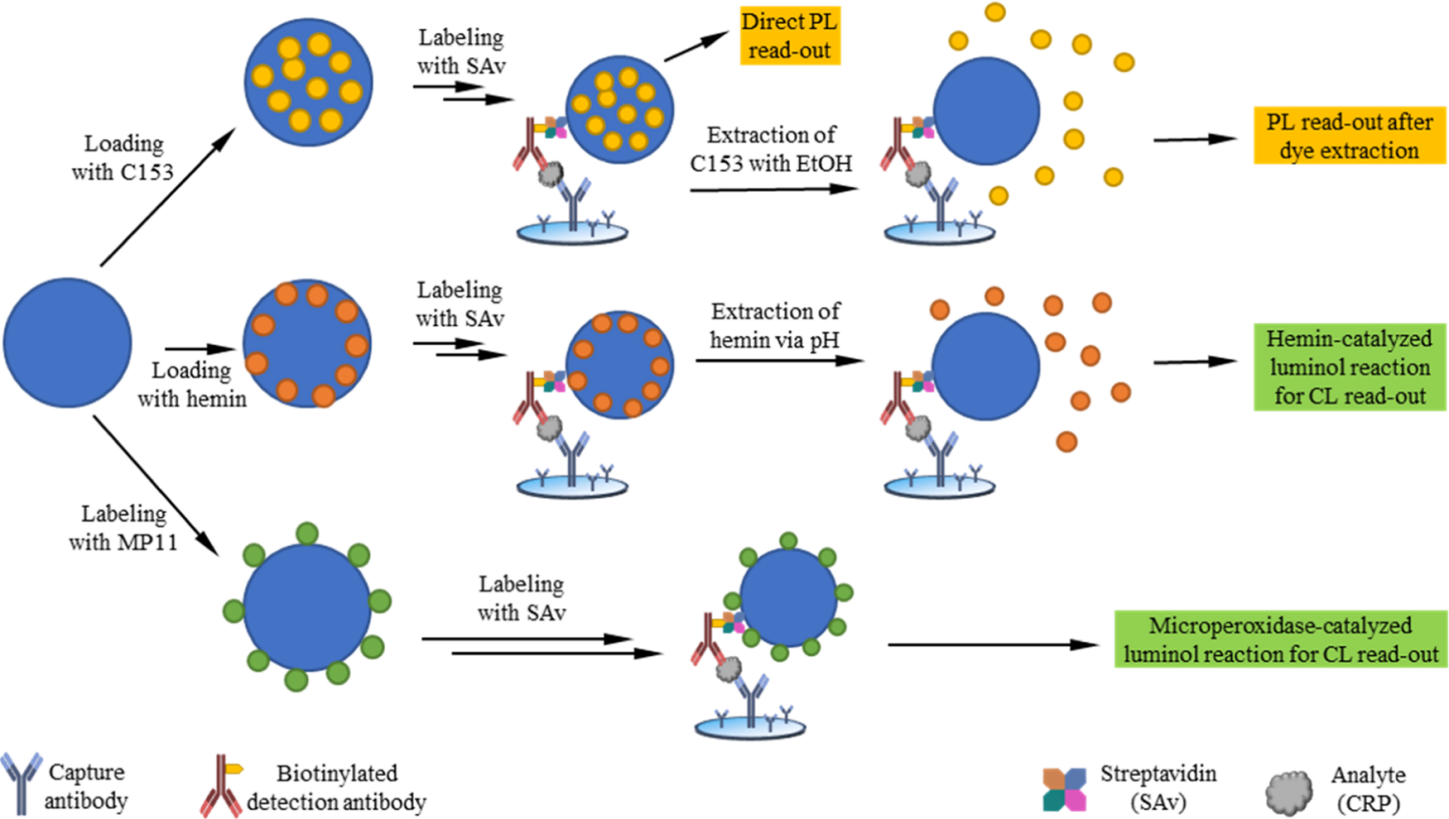
Schematic Presentation of the Heterogeneous Sandwich Immunoassay with 50, 100, 200, and 500 nm Polystyrene Particles (PSP, Blue Spheres) Loaded with (i) the Fluorescent Dye Coumarin 153 (C153; top) or (ii) the Catalyst Hemin (middle), or (iii) labeled with Microperoxidase MP11 (bottom) Utilizing Different Signal Generation Strategies and Photo- and Chemiluminescence (PL; CL) Detection

## Materials and Methods

### Materials

Carboxy-functionalized PSP with nominal sizes of 50, 100, 200, and 500 nm were purchased from Kisker Biotech GmbH. Coumarin 153 (C153) was obtained from Radiant Dyes Laser GmbH. *N*-(3-(Dimethylamino)propyl)-*N*′-ethylcarbodiimide (EDC) hydrochloride, *N*-hydroxysulfosuccinimide sodium salt (sulfo-NHS), biotin-4-fluorescein (B4F), and 2-(*N*-morpholino)ethanesulfonic acid (for MES buffer) were purchased from Sigma-Aldrich GmbH. Sodium chloride, potassium chloride, disodium hydrogen phosphate, and potassium dihydrogen phosphate (for phosphate buffer and phosphate-buffered saline, PBS) as well as sodium carbonate and sodium bicarbonate (for carbonate buffer) were purchased from Carl Roth GmbH. Spectroscopic grade tetrahydrofuran (THF), dimethylformamide (DMF), and ethanol (EtOH) were purchased from Merck KGaA. Tween-20 and sulfuric acid were obtained from AppliChem GmbH. Streptavidin (SAv, 52, 000 g/mol), BCA protein assay kit, horseradish peroxidase-streptavidin conjugate (SAv-HRP), and Turbo-TMB ELISA substrate solution were obtained from Thermo Fisher Scientific, Inc. CRP antigen (30-AC05S) and anti-CRP capture antibodies (ABs) (10-C189B) were purchased from Fitzgerald Industries. Biotinylated anti-CRP detection antibodies (1B-484-C100) were obtained from Exbio Praha. Water was of Milli-Q grade. The 96-well microplates (black, clear, μclear, flat-bottomed chimney wells) for the protein assays were from Greiner Bio-One GmbH. All reagents and solvents were used without further purification.

### General Particle-Related Procedures

All PSP were treated with ultrasound prior to use. Before and after the labeling step, the particles were typically washed three times with Milli-Q water or a certain buffer (as stated below) via centrifugation (Eppendorf 5424R centrifuge; 50 nm: 60 min/24,000*g*; 100 nm: 45 min/21,000*g*; 200 nm: 20 min/8000*g*; 500 nm: 15 min/5000*g*), followed by removal of the supernatant and redispersion in the respective solvent. All labeling and washing steps were carried out at room temperature (RT).

### Preparation of the C153- and Hemin-Loaded PSP

Preparation of the C153- and hemin-loaded PSP was performed following the swelling procedure from Behnke et al. and is further detailed in the Supporting Information (SI).^20,34^ The amount of dye per mg particles was determined via the Beer–Lambert law utilizing the known molar decadic extinction coefficients of C153 (17,600 M^–1^ cm^–1^ in THF) and hemin (64,500 M^–1^ cm^–1^ in DMF) at 414 and 398 nm, respectively.

### Streptavidin Functionalization

Carboxy-functionalized PSP (PS-COOH) were labeled with SAv using EDC/NHS chemistry. Therefore, EDC (32 μL, 150 mM) and sulfo-NHS (16 μL, 300 mM) were added to 800 μL of PS-COOH (50 mg/mL) in MES buffer (0.05 M, pH 5). The mixture was stirred for 1 h and washed with phosphate buffer (10 mM, pH 7.6). Then, SAv (80 μL, 10 mg/mL in phosphate buffer) was added, and the mixture was shaken overnight. Finally, the SAv-labeled particles were washed with water, and the supernatants were kept for SAv quantification. Information about the quantification of SAv can be found in the SI.

### Preparation of MP11-Labeled PSP

Labeling with microperoxidase MP11 was done by adding EDC (4 μL, 150 mM) and sulfo-NHS (2 μL, 300 mM) to 200 μL of unloaded SAv-functionalized PSP (25 mg/mL) in MES buffer (0.05 M, pH 5). The mixture was stirred for 1 h and washed with phosphate buffer (10 mM, pH 7.6). Then, MP11 (20 μL, 10 mg/mL in phosphate buffer) was added, the mixture was shaken for 3 h, and the resulting MP11-SAv-labeled PSP were washed with water.

### CRP Sandwich Immunoassays (ELISA)

All heterogeneous sandwich immunoassays for the determination of C-reactive protein (CRP) were performed in black 96-well clear-bottom, high-binding microplates (Greiner Bio-One) at RT following a standard ELISA protocol, which is outlined in the SI.

### Particle-Based Immunoassays

The particle-based immunoassays were performed analogously to the ELISA, but instead of adding SAv-HRP and Turbo-TMB, the respective SAv-functionalized PSP were added prior to PL or CL detection.

#### PL-Based Detection Using C153-Loaded PSP

50 μL C153-loaded, SAv-functionalized PSP (0.05 mg/mL in blocking buffer) were added to each well, incubated for 30 min, and washed 4× with washing buffer. For the direct read-out of the dye-loaded PSP, 200 μL of washing buffer was added. For read-out after dye extraction from PSP, 200 μL of ethanol were added and the microplate was shaken for 20 min prior to detection. Finally, the emission intensities were detected at 525 nm (excitation at 425 nm) by using the Tecan Infinite M200Pro microplate reader.

#### CL-Based Detection Using Hemin-Loaded PSP

50 μL of hemin-loaded, SAv-functionalized PSP (0.05 mg/mL in blocking buffer) were added to each well and incubated for 30 min. 50 μL of TRIS-carbonate buffer (0.15 M TRIS, 0.06 M carbonate, pH 11) was added to extract hemin from the PSP. Then, 50 μL of Reagent 1 (peroxide solution) and 50 μL of Reagent 2 (luminol solution) of the Pierce ECL Western blotting substrate kit were added well-wise with the Tecan Infinite M200Pro microplate reader an injector. The resulting CL intensities were detected between 380 and 600 nm using photon counting detection.

#### CL-Based Detection Using MP11-Labeled PSP

50 μL of MP11-labeled, SAv-functionalized PSP (0.05 mg/mL in blocking buffer) was added to each well, incubated for 30 min, and the microplate was washed four times with washing buffer. Then, 50 μL of reagent 1 (peroxide solution) and 50 μL of reagent 2 (luminol solution) of the Pierce ECL Western blotting substrate kit were added well-wise using an injector which is part of the Tecan Infinite M200Pro microplate reader. The resulting CL intensities were detected between 380 and 600 nm employing the photon counting technique.

#### Immunoassay Analysis

Due to the partly asymmetric shape of our dose–response curves, five-parameter logistic fits (5PL) were employed for data analysis. The inflection points of the fitted curves (EC50 value) and the limit of detection (LOD), calculated from the mean of the blank and three times its standard deviation, were used as a measure of assay sensitivity. The measurement range was determined as the minimum (*A*_min_) and maximum antigen (*A*_max_) concentration that can be quantitatively measured with respect to the 5PL fit. To compare the different immunoassay formats, the relative dynamic range (RDR) was calculated with eq 1(3)
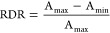
1

## Results and Discussion

Scheme 1 summarizes the different detection concepts for the representative heterogeneous CRP assays and the different parameters explored for signal optimization. As shown in Scheme 1, streptavidin (SAv)-functionalized PSP were loaded with (i) the organic fluorophore C153,^20^ with PL read-out of the reporter-loaded PSP as well as PL detection of the PSP-extracted dye reporter molecules as well as (ii) with hemin and (iii) labeled with MP11. For (ii) and (iii), CL read-out was utilized as another detection method to further optimize assay sensitivity. Therefore, in this screening study, we pursued a step-wise optimization route. First, we examined the influence of the dye staining concentration (1–10 mM) and the two detection schemes (direct read-out vs detection after dye extraction) for 100 nm PSP. Then, we investigated the influence of PSP size (50–500 nm) using the conditions previously found to be optimum. Second, we assessed the influence of parameters such as particle size and reporter loading concentration to derive the advantages and limitations of the different particle-based signal amplification strategies. Subsequently, we optimized the PSP concentration (0.01–0.1 mg/mL) using the PSP size and loading concentration identified before to reveal the best performance (100 nm PSP, 10 mM dye staining). The thereby sequentially derived optimum conditions were finally adopted to the other reporters hemin and MP11 in the final assays using CL as additional detection method known for its high sensitivity to challenge the ELISA assay concept of enzymatic amplification, therefore also performing a CRP ELISA as gold standard. For the final assay comparison, fresh antibodies (from the same batch) kept in the freezer were used; all other parameters, such as temperature and incubation times, were kept constant. Although the extraction/release time can be a crucial factor for our triggered release signal generation concept, we chose to keep the incubation times (including the antibody binding steps and the extraction times used for the release of the signal-generating dyes or the catalyst molecules) always constant, here fixed to 20 min. Thereby, the signal intensities and achievable detection limits are comparable with the time required by a typical ELISA.

### C153-Loaded PSP Reporters

Since most commercial microplate readers used in clinical diagnostics are designed for ELISAs and utilize fixed detection wavelengths of 405 or 450 nm in absorption, fluorescent particle labels should best absorb/emit in this wavelength range. This facilitates reporter implementation into common assay protocols and enables their read-out with commercially available microplate readers. For the exemplary comparison of the direct PL read-out of the dye-loaded PSP and PL read-out after extraction-triggered dye release (Scheme 1, top), we chose the dye C153. C153 exhibits an absorption maximum at 423 nm in ethanol, which lies in the same spectral window as that of the oxidized TMB product used for signal generation in common ELISA (450 nm at pH 1 after addition of 2 M H_2_SO_4_)^35^ and its emission band peaks at 530 nm. In dibutylether (BOB) often used as model system for the PSP matrix, its absorption and fluorescence bands peak at 401 and 470 nm.^20^ Also, C153 is inexpensive, photostable, has a fairly high molar extinction coefficient (e.g., ε_423 nm_ = 18,000 M^–1^ cm^–1^ in ethanol),^36^ high PL quantum yields of Φ_PL_ = 57 and 95% in ethanol and in the PSP,^20^ and a large Stokes shift of about 3500 cm^–1^ (about 199 nm) between its absorption and emission in ethanol minimizing reabsorption. Contrary to most planar dyes with an emission from a localized state like xanthenes, cyanines, and BODIPYs, forming so-called H-type dimers and showing aggregation-induced fluorescence quenching,^22^ C153 has a very low aggregation tendency and did not show fluorescence quenching in PSP even at high dye loading concentrations as previously assessed by us.^20^

The surface of the C153-stained PSP was then functionalized with streptavidin (SAv) for binding to the biotinylated detection antibodies after the formation of a sandwich between the immobilized capture antibodies and CRP antigen. Two detection schemes were used for the C153-loaded PSP particles, the direct read-out of the PL intensity of the dye-loaded PSP and PL detection of C153 released from the PSP triggered by ethanol. For both detection schemes, optimization of the PL signal intensity should be achievable by varying the particle size and dye loading concentration, as subsequently assessed.

**Figure 1:**
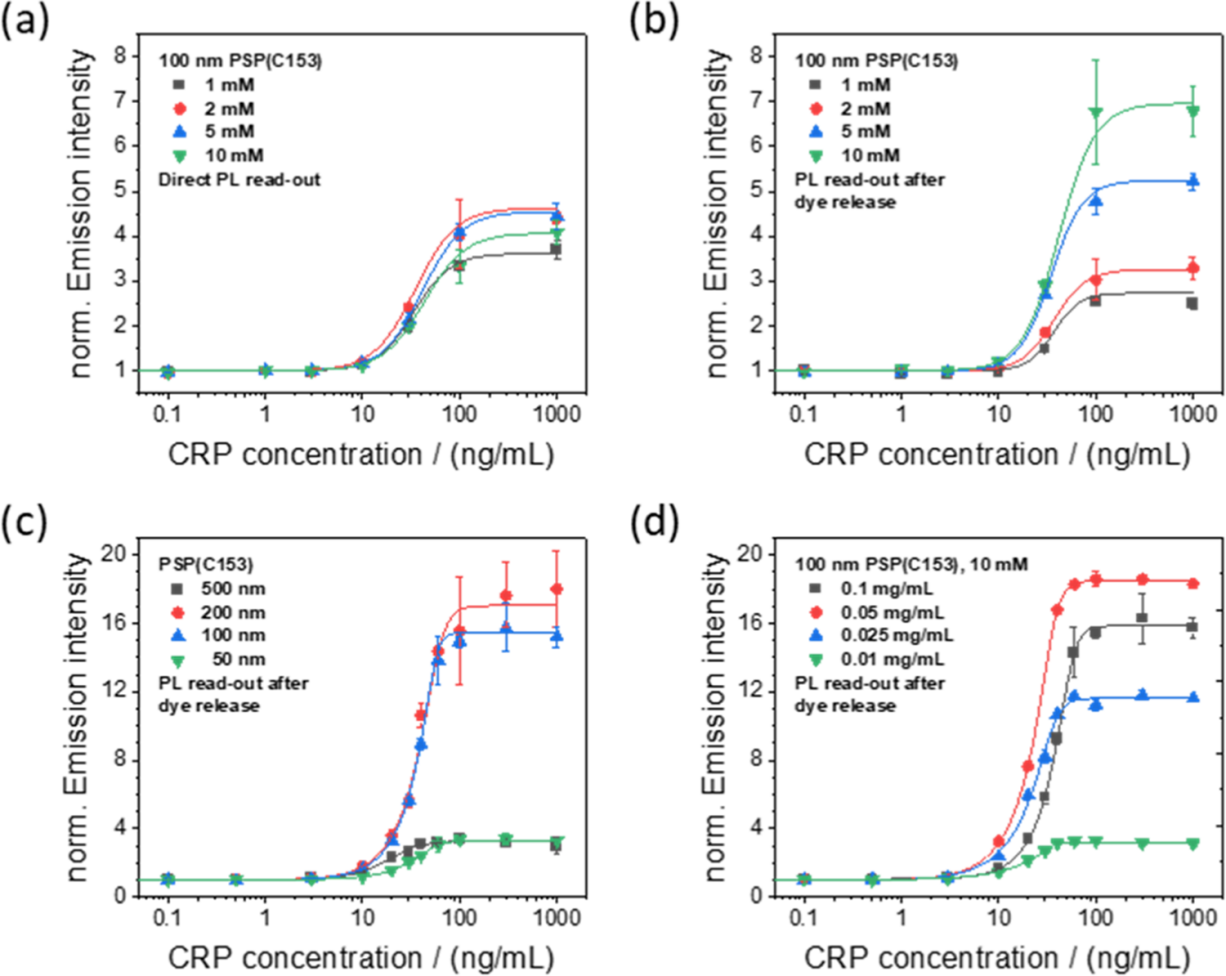
Dose–response curves obtained for the heterogeneous sandwich CRP immunoassays using C153-loaded PSP. The measured emission intensities are normalized to the respective background signal for each assay. These intensities were recorded by measuring (a) the PL intensity of the dye-loaded PSP or (b) the PL signals of the dye molecules released from the particles triggered by extraction with EtOH using 0.1 mg/mL PSP in each experiment. The performance of the differently sized C153-loaded PSP reporters with a PSP concentration of 0.1 mg/mL and different concentrations of 100 nm sized PSP, stained with 10 mM C153 in the CRP immunoassay measured under otherwise identical conditions, is shown in (c) and (d), respectively.

To representatively determine the optimum PSP size for our particle-based immunoassays, SAv-functionalized C153-loaded PSP of different sizes (50, 100, 200, and 500 nm) prepared using C153 concentrations of 1–10 mM, were characterized regarding their spectroscopic properties, and compared with respect to their performance as reporters in the CRP assay. First, for 100 nm SAv-functionalized PSP stained with different C153 concentrations, the mean number of dyes per particle was determined. The obtained results, shown in Figure S1, revealed a nearly linear signal increase for the smaller C153 staining concentrations of 1–5 mM. However, when increasing the staining concentration from 5 mM to 10 mM, the number of dyes per PSP did not further linearly increase. This suggests the onset of dye saturation of the polymer matrix in this concentration range.^34^ The determination of the number of surface-bound SAv molecules revealed a close match for all C153-loaded PSP. This confirms that SAv surface labeling is not affected by the number of incorporated dyes (Table S1). DLS measurements revealed that the increase in the dye staining concentration did not affect the hydrodynamic diameter of the PSP. After functionalization with SAv, the PSP size increased by 15 nm, demonstrating the successful immobilization of SAv on the PSP surface (see Table S2). Subsequently, the performance of the different 100 nm C153-loaded SAv-functionalized particles in the CRP immunoassay was compared using the direct PL read-out approach. The relatively similar intensity of the PL signals with increasing CRP antigen concentration (Figure 1a) reveals that the measured PL intensities do not correlate with the actual number of dyes per particle. A maximum PL intensity is already reached for PSP made with a C153 staining concentration of 2 mM. Higher dye concentrations can even result in a decreased overall PL intensity, possibly due to inner filter effects within the stained PSP, hampering the excitation of C153 molecules in the inner particle core. The comparison of the slopes reveals a similar dynamic range for all C153-stained PSP independent of the dye staining concentration used, as to be expected from the nearly identical SAv number per particle for our PSP reporters. Nevertheless, the steeper slope for PSP particles stained with 2 mM C153 points to a higher sensitivity for these NP reporters compared with the other particles.

For the extraction-triggered dye release, the PSP loaded with a higher dye concentration are expected to yield higher PL signals since with this approach PL reducing effects like inner filter effects, reabsorption, and dye–dye interactions can be circumvented. Dye release was initiated by the addition of ethanol. The PL signals were measured 20 min after ethanol addition, when dye release was completed as shown for a representative sample. As shown in Figure 1b, the PL signal intensities obtained in the CRP immunoassay correlate well with the number of dyes per PSP. This confirms a successful signal amplification by our dye release approach. For this signal generation concept, the achievable PL intensities are only limited by the dye loading capacity of the 100 nm PSP. Principally, the amount of released dyes can be increased by using larger particles, as the dye number per PSP directly depends on the particle volume (Table S3). Therefore, different PSP sizes ranging from 50 to 500 nm were tested in the CRP immunoassay under identical conditions. The results are summarized in Figure 1c and Table S4. The dose–response curves reveal that particle sizes of 100 and 200 nm yield similar results and exceed the performance of the smallest and the largest PSP examined in this study, i.e., 50 and 500 nm PSP. We ascribe the weak performance of the 50 nm PSP to the lower number of reporter dyes within the PSP.

A possible explanation for the weaker performance of the 500 nm PSP could be a less efficient, i.e., nonquantitative dye release within the extraction time of 20 min, which we chose to be comparable to the ELISA. Moreover, the larger PSP size can potentially also result in a competition for the binding to the biotinylated detection antibodies leading to an overall low number of PSP per well and thus a lower PL intensity after dye extraction. A more detailed investigation of these assumptions was beyond the scope of this first screening study. As an increase in PSP size apparently did not automatically lead to higher PL signals for particle reporters with sizes >200 nm, we examined another possibility of a straightforward PL enhancement, i.e., the increase of the concentration of the C153-stained PSP, to further optimize the performance of the CRP assay. The results obtained with different concentrations of 100 nm C153-stained PSP in the CRP assay are summarized in Figure 1d. As shown in this figure, the signal intensity increased with increasing PSP concentration, and the measured PL intensity could be further enhanced. The signal amplification, however, does not linearly depend on the PSP concentration.

### Hemin-Loaded PSP Reporters

To further push the CRP assay performance with PSP reporters to its limits, we extended our PSP loading and release concept to an inexpensive and long-term stable nonenzymatic catalyst, here hemin. This allows for the exploitation of the hemin-catalyzed luminol reaction for the generation of a chemiluminescence (CL) signal (Scheme 1, middle), which can be read out with the same microplate reader as the PL-based CRP immunoassays. Also with this approach, a possible influence of the particle size distribution on analyte quantification can be elegantly avoided, as well as a possible influence of the particle matrix on the spectroscopic features of the signal-generating reporters. In addition to providing a straightforward parameter for signal amplification for our release-based signaling strategies, this detection scheme can be realized with simple and inexpensive instrumentation, as CL does not require an excitation light source. For the CL approach, the intensity of the read-out optical signals should solely depend on the stability of the catalyst, the efficiency of catalyst loading, and the efficiency of catalyst release from the carrier particles. Thus, we can expect similar results when increasing the loading amount of hemin for PSP of identical size. Therefore, for this enhancement strategy, we focused on optimizing the effect of concentration of hemin molecules released from our carrier particles on the intensity of the recorded CL signals, here by adjusting the parameters of PSP size and PSP hemin loading concentration. As shown in Table S5, the overall hemin amount increased with increasing particle size using a constant loading concentration of 10 mM hemin.

**Figure 2:**
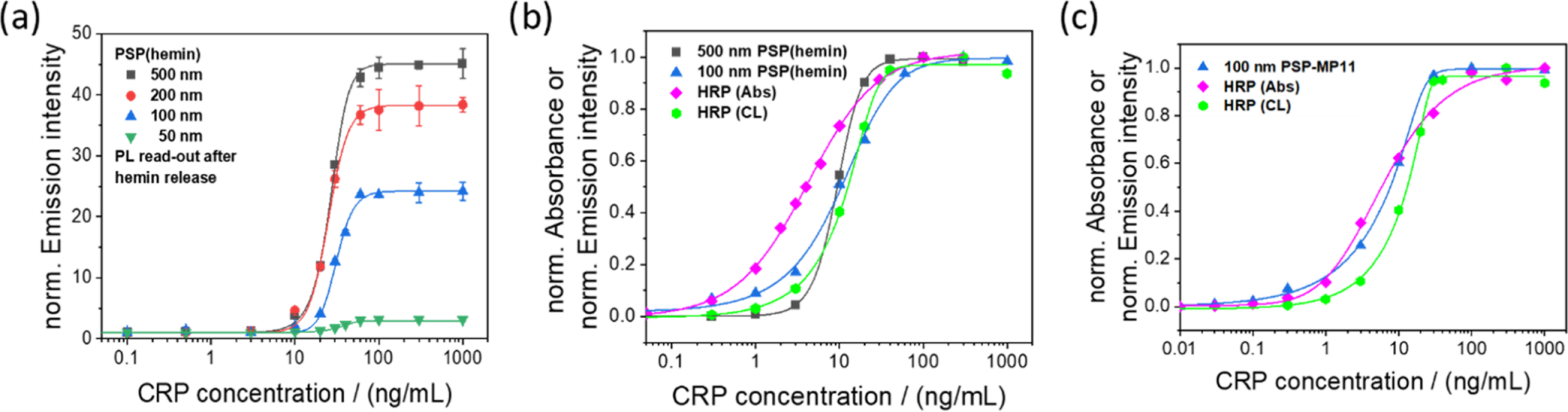
Dose–response curves of the heterogeneous sandwich CRP immunoassays obtained with hemin-loaded PSP of different sizes (a) determined for a PSP concentration of 0.05 mg/mL. (b) Optimized CRP immunoassays with 100 and 500 nm hemin-loaded PSP in comparison to a conventional HRP-based ELISA utilizing the luminol reaction with CL read-out and the frequently used HRP substrate TMB with absorbance read-out (Abs). (c) Dose–response curves obtained for CRP immunoassays with MP11-labeled PSP in comparison to the HRP-based ELISA, utilizing either the luminol reaction with chemiluminescence (CL) read-out or the common HRP substrate TMB with absorbance read-out (Abs).

A comparison of the CL signals that were obtained with differently sized hemin-loaded PSP after extraction-triggered release of hemin, catalyzing the signal-generating luminol reaction, is highlighted in Figure 2. Figure 2a reveals a similar performance of 100 and 200 nm hemin-loaded PSP particles and a low CL signal for the 50 nm PSP particles. The latter is ascribed to the low hemin-loading capacity of the small PSP. The highest CL signal was obtained for 500 nm hemin-loaded PSP. Like before, the CL enhancement is not linearly correlated to the particle size and PSP hemin loading capacity. A comparison of the optimized CL assays utilizing 100 and 500 nm hemin-loaded PSP with the gold standard ELISA confirms a similar performance of all CRP assays (Figure 2b). Major differences between the differently sized hemin-loaded PSP follow from the assay dynamic range. Using 500 nm hemin-loaded PSP led to a very steep slope, indicating a high sensitivity in a small CRP concentration range. This limits the assay’s applicability compared to a CL-based ELISA or 100 nm hemin-loaded PSP. The sigmoidal response curve of 100 nm hemin-loaded PSP is very similar to that of the CL-based ELISA assay, but the conventional ELISA still shows a higher sensitivity than all our CL-based release approaches.

### Microperoxidase-Labeled PSP Reporter

Alternatively, for PSP staining, catalyst molecules can be bound to the particle surface by established conjugation chemistries. This renders the catalyst easily accessible for catalyzing the signal-producing CL reaction (Scheme 1, bottom). In this case, the particles act as scaffolds that allow for high catalyst numbers due to the large particle surface, while a time-consuming release step is avoided. This approach also prevents an influence of the particle size distribution on the signal intensity and quantification. To explore the potential of this signal enhancement approach, we chose well-known microperoxidase MP11 (MP11) as a catalyst which was then immobilized on the surface of 100 nm PSP, previously functionalized with SAv. The chemical structure of MP11 bound to the PSP is shown in Figure S2. A comparison of the performance of the MP11-labeled PSP with that of the ELISA assay is shown in Figure 2c. Although both assays rely on the same luminol reaction for CL generation, the MP11-decorated PSP show a larger dynamic range and a higher sensitivity. Also, the results of the MP11-labeled PSP are in a similar range as the results obtained with the widely utilized colorimetric ELISA with the HRP substrate TMB, which was used as a benchmark for this study. This makes our MP11-decorated PSP a realistic competitor of the gold standard ELISA.

### Comparison of the Signal Amplification Strategies

To compare achievable sensitivities of EIA and FIA, we evaluated the test midpoint (EC50 value), linear measurement range, and relative dynamic range (RDR) for each assay (Table 1). The test midpoint, known as the point of inflection of the sigmoidally fitted dose–response curve, is a measure of assay sensitivity. The linear measurement range should be broad to enable the detection of a larger variety of antigen concentrations using the same assay. As a measure for assay comparison, exploiting different detection schemes such as absorbance, PL, and CL, we used the relative dynamic range. Under these circumstances, a value ≥0.90 is desired to allow the precise quantification of an unknown antigen concentration. The larger EC50 values of the assays relying on the extraction-triggered release of C153 and PL read-out reveal the lower sensitivity of this approach in comparison to the other amplification strategies. Possible reasons are a lower detection sensitivity of the instrument used for assay read-out and/or a poorer signal-to-noise ratio for very small concentrations of such an organic dye. As follows from the comparison of the detection sensitivities of the different enhancement approaches utilizing CL, the CRP immunoassay with MP11-labeled PSP is clearly the most sensitive one. Thereby, a performance that is very close to that of the colorimetric “gold standard” ELISA could be achieved. Regarding the RDR evaluation, only assays utilizing CL and absorbance detection could reach in part values of about 0.9. The 500 nm PSP reporters yielded a higher sensitivity but a lower RDR compared to 100 nm PSP for identical extraction times triggering reporter release. Encouragingly, usage of the MP11-labeled PSP led to a higher RDR than the RDR obtained for the ELISA with CL readout, and the RDR value realized with the former signal amplification strategy is comparable to the performance of the photometric ELISA. To better evaluate the performance of the CRP immunoassay with the MP11-labeled PSP and CL detection and the CL-based ELISA, note that we performed the CL-based assays and the luminol reaction under the same conditions as the other assays with the PSP reporters. This was done as the goal of this study was a comparison of the different release-triggered signal generation and detection concepts and not an optimization of the CL-ELISA, but an optimization of our own assays.

**Table 1:** Overview of the EC50 Values, Limits of Detection (LOD), Measurement Range, and Relative Dynamic Range (RDR) Obtained for the Different Detection Schemes Using Photoluminescence (PL), Chemiluminescence (CL), or Absorbance (Abs) Measurements

assay type	detection scheme	particle size (nm)	EC50 (ng/mL)	LOD (ng/mL)	measurement range (ng/mL)	Rel. dynamic range (RDR)
dye-loaded PSP	C153 (PL)	100	23.7 ± 0.4	4.9	12–45	0.73
		500	18.3 ± 0.8	7.2	16–34	0.53
hem-loaded PSP	luminol (CL)	100	10.9 ± 0.6	1.1	2.7–41	0.93
		500	9.5 ± 0.9	2.0	4.5–20	0.78
MP11-labeled PSP	luminol (CL)	100	7.6 ± 0.3	0.10	2–28	0.93
ELISA	HRP + luminol (CL)		12.5 ± 0.5	1.4	5–30	0.83
	HRP + TMB (Abs)		5.8 ± 0.7	0.13	0.74–46	0.98

## Conclusions and Outlook

Aiming for the development of simple assay amplification schemes, in this sequential screening study, we explored different particle-based signal amplification strategies for heterogeneous sandwich immunoassays. Polystyrene particles (PSP) assessed together with an extraction-triggered release step included streptavidin (SAv)-functionalized PSP loaded with the dye coumarin 153 (C153) for photoluminescence (PL) read-out and hemin molecules catalyzing the luminol reaction for chemiluminescence (CL) detection. As a third approach, PSP surface-labeled with hemin-based microperoxidase MP11, also catalyzing the luminol reaction and CL read-out, were examined to further simplify the assay and reduce the number of work steps. For all 3 PSP types and detection schemes, parameters like particle size, reporter loading concentration, and PSP surface chemistry were studied. PSP, optimized for each of the amplification approaches, were then applied in a heterogeneous sandwich immunoassay for the inflammation marker C-reactive protein (CRP) and their performance was compared to the performance of an established photometric ELISA. Thereby, the incubation times (including antibody binding steps and extraction times for reporter release) were kept constant at a time frame of 20 min. We could demonstrate the advantage of our extraction-triggered release approach by comparing the PL read-out after dye release and PL detection of the intact C153-stained PSP. This simple and fast extraction-triggered release step circumvented otherwise faced limitations for larger dye loading concentrations like inner filter effects and dye–dye interactions, thus improving assay sensitivity. However, the PL read-out approach was not as sensitive as the other signal enhancement strategies explored in this screening study. Combining hemin-loaded carrier beads, extraction-triggered hemin release, and CL detection provided sensitivities closely approaching that of a photometric ELISA. Also, the signal intensity was independent of PSP size and only controlled by the concentration of the released hemin. Though a trend could be clearly seen toward smaller EC50 (test midpoint) values for larger-sized PSP and hence to a higher sensitivity, the limits of detection (LOD) and relative dynamic range (RDR) obtained with the 100-nm-sized PSP reporters were superior to the behavior of the 500-nm-sized PSP. The finding that larger PSP yield a smaller RDR range suggests a potentially too short extraction time frame for the largest size PSP and/or a steric hindrance to the binding of the immobilized detection antibodies. As a promising and simple signal enhancement strategy, we identified MP11-labeled PSP yielding EC50 values and an RDR in the CRP assay, closely comparable with the photometric ELISA. This indicates that our amplification schemes utilizing CL can principally compete with the ELISA.

In the future, we plan to further boost the performance of CL-based amplification schemes. For example, we will explore the applicability of a cleavable linker for the controlled release of MP11 from the PSP surface and the suitability of other carriers such as mesoporous silica particles, which allow for the incorporation of a larger number of MP11.

## Supporting Information Available

Extended method part, quantitative information of the C153 per NP for the staining concentrations, overview tables about the SAv/particle and C153 dyes/particle, hemin per particle ratio, all in dependence of the particle size, and the chemical structure of MP11 bound to the PSP (PDF)
