# Supplementary material for: Exploring Simple Particle-Based Signal Amplification Strategies in a Heterogeneous Sandwich Immunoassay with Optical Detection
Source: Anal Chem. 2024 Mar 18;96(13):5078–85. doi: 10.1021/acs.analchem.3c03691 (PMC10993196; doi:10.1021/acs.analchem.3c03691)
Supplement: Supplementary file 1 — ac3c03691_si_001.pdf [file ac3c03691_si_001.pdf]

Supporting Information

Exploring simple particle-based signal amplification strategies in a heterogeneous sandwich immunoassay with optical detection

Daniel Geißler[#], K. David Wegner[#], Christin Fischer[†], and Ute Resch-Genger[*]

Federal Institute for Materials Research and Testing (BAM), Division *Biophotonics*, Richard-Willstaetter-Str. 11, 12489 Berlin, Germany.

*E-mail: ute.resch@bam.de. Phone: +49 / (0)30 / 8104-1134.

#: both authors contributed equally

†M.Sc. Christin Fischer, christin_fischer88@t-online.de

Table of Content

Methods 2

Figure S1: Mean number of C153 dyes per 100 nm particle after staining with different dye concentrations. 2

Table S1: Mean number of SAv molecules per particle determined with the BCA assay and biotin-4-fluorescein (B4F) titration. 2

Table S2: PSP diameters obtained by DLS measurements of 100 nm PSP using different dye staining concentrations and before and after SAv functionalization. 3

Table S3: Mean number of C153 dyes per particle for different particle sizes after staining with 10 mM C153, and mean number of SAv molecules per particle as measured with BCA assay and Biotin-4-Fluorescein (B4F) titration. 3

Table S4: EC50 values of differently sized dye-stained PSP beads (0.05 mg/ mL) using the direct PL read-out (in PSP) and after dissolution of the PSP beads (in EtOH). 3

Table S5: Mean number of hemin molecules per particle for different particle sizes after PSP staining with 10 mM hemin, and mean number of SAv molecules per hemin-loaded PSP determined with the BCA assay and biotin-4-fluorescein (B4F) titration. 3

Figure S2: Chemical structure of microperoxidase MP11 (MP11) bound to the surface of carboxylated PSP. 4

REFERENCES 4

Methods

Preparation of the C153- and hemin loaded PSP. Briefly, 100 µL of C153 dissolved in THF (1-10 mM) or hemin dissolved in DMF (1-5 mM) were added to 600 µL of a PSP dispersion in water (10 mg/mL) and the mixture was shaken for 30 min at RT. Then, 800 µL water were added and the particles were washed three times with water, thereby adjusting the particle concentration to 50 mg/mL. The amount of incorporated C153 or hemin was determined by adding 200 µL of THF or DMF to 20 µL of the PSP dispersion (1 mg particles) to dissolve the particles and release the payload. The resulting solutions were diluted with THF or DMF to an appropriate optical density and the absorbance was measured at 414 nm (C153) or 398 nm (hemin).

Streptavidin quantification. SAv was quantified with the bicinchoninic acid (BCA) assay and biotin-4-fluorescein (B4F) titration in the supernatants of the SAv labeling reactions (unbound SAv) and in the SAv-labeled PSP dispersions (bound SAv) to obtain mass balances. The BCA assays were performed according to the manufacturer instructions and the B4F titrations were carried out following the protocols published by Quevedo *et al.*.[1] Please note that with the BCA assay, all proteins, i.e., SAv molecules, present are detected while with the B4F assay, only the accessible binding pockets of the SAv molecules are determined. This number is usually lower than 4 per SAv molecule. [2] In addition, this number could be affected by steric factors related to PSP size that may also differ between the differently sized PSP explored. However, for a comparison of the assay results obtained with the differently sized PSP, an absolutely SAv quantification was not required.

CRP sandwich immunoassays (ELISA). Briefly, 50 µL anti-CRP capture AB (1 µg/mL in 0.05 M carbonate buffer, pH 9.6) were added to the wells, immobilized for 2 h, and washed 4x with washing buffer (PBS buffer with 0.05% Tween-20). Next, 300 µL blocking buffer (washing buffer with 10 mg/mL BSA) were added, immobilized for 30 min, and washed four times with washing buffer. Then, 50 µL of the CRP standards (0 – 1,000 ng/mL CRP in blocking buffer) were added, incubated for 1 h, and washed 4x with washing buffer. Finally, 50 µL of the biotinylated anti-CRP detection AB (0.5 µg/mL in blocking buffer) were added, incubated for 1 h, and washed 4x with washing buffer. All washing steps were performed with a HydroFlex™ (Tecan Trading AG, Switzerland) microplate washer.

For the standard ELISA with absorption read-out, 50 µL SAv-HRP (0.1 µg/mL in blocking buffer) were added to each well, incubated for 1 h, and the microplate washed 4x with washing buffer. Next, 100 µL Turbo TMB-ELISA substrate solution were added. After 30 min, 100 µL sulfuric acid (2 M) were added. The absorbance at 450 nm was detected with the Tecan Infinite M200Pro microplate reader (Tecan Trading AG, Switzerland; top reading).

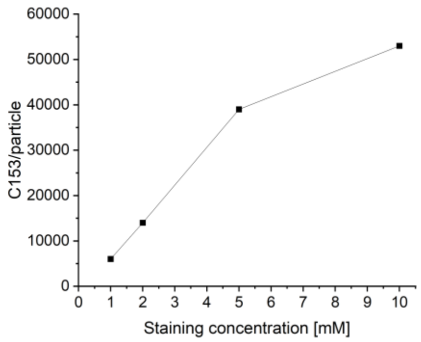

Figure S1: Mean number of C153 dyes per 100 nm particle after staining with different dye concentrations.

Table S1: Mean number of SAv molecules per particle determined with the BCA assay and biotin-4-fluorescein (B4F) titration.

Staining conc. (mM)	SAv/particle (BCA / B4F)
1	51 / 32
2	53 / 41
5	52 / 34
10	56 / 38

Table S2: PSP diameters obtained by DLS measurements of 100 nm PSP using different dye staining concentrations and before and after SAv functionalization.

Dye conc. [mmol/L]	Before functionalization		After functionalization	
	Z-average (nm)	PDI	Z-average (nm)	PDI
1	117.9±0.6	0.009±0.010	134.0±0.9	0.018±0.012
2	119.2±1.1	0.011±0.008	133.1±1.4	0.021±0.009
5	118.1±0.5	0.008±0.07	132.9±0.7	0.015±0.011
10	118.3±0.9	0.009±0.011	133.7±2.3	0.018±0.007

Table S3: Mean number of C153 dyes per particle for different particle sizes after staining with 10 mM C153, and mean number of SAv molecules per particle as measured with BCA assay and Biotin-4-Fluorescein (B4F) titration.

Particle size (nm)	C153 dyes per particle	SAv/particle (BCA / B4F)
50	4,500	16 / 13
100	53,000	115 / 63
200	280,000	390 / 260
500	4,500,000	70,000 / 7600

Table S4: EC50 values of differently sized dye-stained PSP beads (0.05 mg/ mL) using the direct PL read-out (in PSP) and after dissolution of the PSP beads (in EtOH).

Particle size (nm)	Read out condition	EC50 value [ng/mL]
50	in PSP	29.5
	in EtOH	24.3
100	in PSP	24.8
	in EtOH	23.7
200	in PSP	21.8
	in EtOH	23.5
500	in PSP	18.8
	in EtOH	18.3

Table S5: Mean number of hemin molecules per particle for different particle sizes after PSP staining with 10 mM hemin, and mean number of SAv molecules per hemin-loaded PSP determined with the BCA assay and biotin-4-fluorescein (B4F) titration.

Particle size (nm)	Hemin per particle	SAv/particle (BCA / B4F)
50	1,000	16 / 15
100	22,000	126 / 77
200	115,000	730 / 750
500	1,500,000	42,000 / 9100

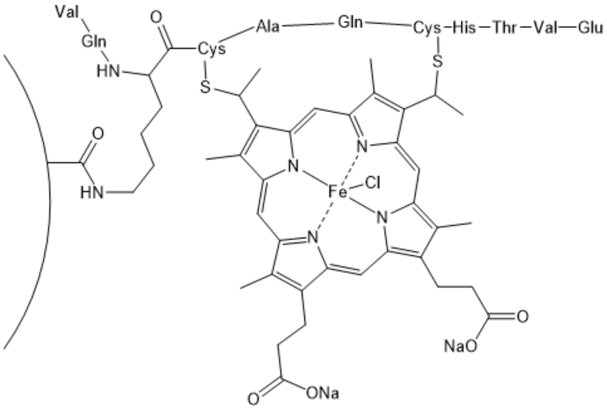

Figure S2: Chemical structure of microperoxidase MP11 (MP11) bound to the surface of carboxylated PSP.

REFERENCES

(1) Quevedo, P. D.; Behnke, T.; Resch-Genger, U. Streptavidin conjugation and quantification-a method evaluation for nanoparticles. *Anal. Bioanal. Chem.* **2016**, *408* (15), 4133-4149.

(2) Schiestel, T.; Brunner, H.; Tovar, G. E. M. Controlled surface functionalization of silica nanospheres by covalent conjugation reactions and preparation of high density streptavidin nanoparticles. *J. Nanosci. Nanotechnol.* **2004**, *4* (5), 504-511.
